# Trastuzumab deruxtecan (DS-8201) plus bevacizumab for HER2-low metastatic breast cancer with malignant pleural effusion: a case report and literature review

**DOI:** 10.3389/fonc.2025.1694553

**Published:** 2026-01-12

**Authors:** Hao Han, Jing Liu, Congcong Wang, Miaomiao Yang, Jiannan Liu, Ping Sun

**Affiliations:** 1 Affiliated Hospital of Shandong Second Medical University, School of Clinical Medicine, Shandong Second Medical University, Weifang, Shandong, China; 2 Department of Pathology, The Affiliated Yantai Yuhuangding Hospital of Qingdao University, Yantai, Shandong, China; 3 Department of Oncology, The Affiliated Yantai Yuhuangding Hospital of Qingdao University, Yantai, Shandong, China

**Keywords:** bevacizumab, case report, DS-8201, Her2-low breast cancer, pleural effusion

## Abstract

**Introduction:**

HER2-low breast cancer has emerged as a distinct molecular subtype with unique biological features and therapeutic significance. Trastuzumab deruxtecan, an antibody-drug conjugate, has shown promising efficacy in both HER2-high and HER2-low disease.

**Case description:**

We report the case of a 69-year-old woman with a 17-year history of breast cancer, initially diagnosed in 2007 with invasive ductal carcinoma and lymph node metastasis. After multiple lines of systemic therapy, the disease progressed with dynamic reduction of HER2 expression from 3+ to 1+, ultimately confirming HER2-low advanced breast cancer. Recurrent malignant pleural effusion became the predominant manifestation, severely impairing quality of life.

**Intervention and outcomes:**

DS-8201 monotherapy was initiated but failed to control pleural effusion. Sequential combinations of DS-8201 with intrapleural cisplatin and then intrapleural bevacizumab were attempted, with only the latter yielding significant benefit. The regimen was subsequently optimized to intravenous DS-8201 plus bevacizumab, resulting in rapid symptom relief, substantial reduction of pleural effusion, and sustained disease control. At the latest follow-up, the patient achieved 18 months of stable disease with improved quality of life and no severe adverse events.

**Conclusion:**

To our knowledge, this is the first reported case of DS-8201 plus bevacizumab for HER2-low metastatic breast cancer with malignant pleural effusion. This case highlights the potential of this regimen as a promising therapeutic option for patients with limited alternatives.

## Introduction

Breast cancer remains the most prevalent malignancy among women worldwide. Despite advances in early detection and treatment, its global incidence continues to rise, posing a significant public health challenge. While targeted therapy, endocrine therapy, and immunotherapy have improved prognosis for some patients, treatment options remain limited for metastatic breast cancer patients progressing after multiple systemic treatments. HER2-low breast cancer has emerged in recent years as a biologically and therapeutically distinct subtype, lying between the traditional classifications of HER2-positive and HER2-negative disease. It is characterized by an HER2 immunohistochemistry (IHC) score of 1+ or 2+ with negative *in situ* hybridization (ISH) testing (1). Traditional chemotherapy has shown limited efficacy in this subtype, and the overall prognosis tends to be unfavorable. Consequently, the search for more targeted treatment strategies has become a key focus in clinical practice. The advent of antibody-drug conjugates has opened up new therapeutic avenues for patients with this subtype. Antibody-drug conjugates (ADCs) offer a novel therapeutic avenue by delivering cytotoxic agents precisely to tumor cells, enhancing efficacy while reducing systemic toxicity (2). The landmark DESTINY-Breast04 trial (3) was the first to demonstrate the significant efficacy of DS-8201 in patients with HER2-low cancer of the breast, offering a brand-new therapeutic alternative for this subgroup. The DESTINY-Breast06 trial (4) extended DS-8201’s role to hormone receptor-positive (HR+) HER2-low or HER2-ultralow patients, and its positive results led to FDA approval for this population, representing an important advancement in precision HER2-targeted therapy. For HR-negative (HR-) HER2-low breast cancer, effectively triple-negative breast cancer (TNBC), the Trop-2 targeting ADC sacituzumab govitecan is preferred, based on the ASCENT trial’s (5) demonstration of improved progression-free survival (PFS) and overall survival (OS) compared to chemotherapy. Therefore, HR status is critical in selecting ADC therapies in HER2-low breast cancer: DS-8201 for HR+ patients, sacituzumab govitecan for HR- patients. Bevacizumab is a recombinant humanized IgG1 monoclonal antibody that can specifically target vascular endothelial growth factor (VEGF), block its binding to the receptor, and thereby inhibiting tumor angiogenesis, reducing vascular permeability, improving the hypoxic state in tumor tissues, and enhancing the delivery efficiency of drugs in tumor tissues (6). In several key trials such as E2100 (7), AVADO (8), and RIBBON-1 (9), the combination of bevacizumab with chemotherapy significantly boosted PFS for patients with HER2-negative. As there was no significant benefit in subsequent OS but the toxicity increased significantly, the FDA withdrew its approval for the therapy of breast cancer in 2011. However, subsequent studies still show that bevacizumab has certain clinical value in controlling malignant effusion caused by tumors (10, 11), especially in advanced patients with pleural effusion or ascites as the main manifestation, it can play a positive role in alleviating discomfort and enhancing the standard of life.

Malignant pleural effusion is common in advanced breast cancer with pleural metastasis, severely impacting quality of life and signaling poor prognosis. No effective therapies specifically target HER2-low breast cancer with predominant pleural effusion, representing a clinical challenge. This report presents a HER2-low breast cancer case with recurrent malignant pleural effusion, refractory to multiple lines of therapy and traditional chemotherapy. Treatment with DS-8201 plus bevacizumab achieved favorable clinical response, offering a novel therapeutic approach.

## Case presentation

A 69-year-old female patient was first diagnosed with breast cancer in February 2007 after presenting with a painless lump in the left breast for three months. The patient had no prior comorbidities and no family history of breast cancer. The patient’s left breast cancer was treated with a modified radical mastectomy. Postoperative pathology revealed invasive ductal carcinoma involving the nipple, with lymphovascular invasion, and metastasis in 3 out of 17 axillary lymph nodes. The postoperative stage was pT1cN1aM0. IHC showed ER (+), PR (+), and HER2 (3+). Following surgery, the patient underwent eight cycles of adjuvant chemotherapy with doxorubicin (60 mg/m², once every 21 days) combined with cyclophosphamide (600 mg/m², once every 21 days), succeeded by sequential paclitaxel (175 mg/m², once every 21 days). The patient refused trastuzumab targeted therapy due to financial constraints. Given the patient’s premenopausal, HR+ status and the 2007 clinical guideline recommendations—during which gonadotropin-releasing hormone agonist plus aromatase inhibitor was not a standard therapy—oral tamoxifen (10 mg, twice daily) was selected for endocrine therapy, and the disease remained well controlled. Subsequent follow-up was conducted regularly, and until November 2012, disease progression was observed, with a disease-free survival of 69 months.

In November 2012, left supraclavicular lymph node metastasis was detected. For this metastatic event, the patient was treated at Beijing Shijitan Hospital with trastuzumab (initial loading dose of 8 mg/kg, followed by 6 mg/kg, every 21 days) plus gemcitabine (1250 mg/m² on days 1 and 8, every 21 days) for six cycles of chemotherapy. Concurrent radiotherapy was delivered to the left supraclavicular lymph node region, targeting the planning gross tumor volume (PGTV), with 57.5–60 Gy/25f. Following the completion of this intensive phase in Beijing, the patient returned to our institution and began long-term maintenance therapy with trastuzumab (6 mg/kg, once every 21 days) plus anastrozole (6 mg/kg, once every 21 days), given the postmenopausal, HR+ status and the achieved disease control. The disease remained stable during this phase of treatment, and until August 2016, disease progression was observed again, with a progression-free survival (PFS) of 45 months.

In August 2016, a mass reappeared in the left axilla. Core needle biopsy confirmed recurrent metastatic breast cancer. IHC showed ER (10%+), PR (approximately 2% weak +), HER2 (3+), CK5/6 (−), P63 (−), GATA-3 (+), EMA (partially +), and Ki-67 proliferation index of about 50%. Based on the continued use of trastuzumab, the chemotherapy regimen was changed to vinorelbine (25 mg/m², on days 1 and 8, every 21 days) in combination for four cycles. Considering that the patient had previously received long-term anastrozole therapy and might have developed resistance to the aromatase inhibitor after this recurrence, toremifene (60 mg, once daily) was used for maintenance treatment instead. The disease remained stable during this phase of treatment, with the PFS of 12 months.

In August 2017, the left axillary lesion had a repeat core needle biopsy and revealed characteristics of mucinous cancer. IHC demonstrated ER (approximately 20% moderate-strong+), PR (approximately 30% weak-moderate+), HER2 (3+), CK5/6 (−), P63 (−), GATA-3 (+), EMA (partial+), and Ki-67 proliferation index of about 40%. Subsequently, the patient was treated with lapatinib (1250mg, once daily) plus capecitabine (1000mg/m2, twice daily on days 1–14 of each 21-day cycle), achieving stable disease until 2019. Pyrotinib is a second-generation oral irreversible HER2-targeted drug independently developed in China, characterized by multi-target activity, high efficacy, low resistance, and good safety. Since its approval in 2020, due to its affordable price and high accessibility, the patient’s treatment regimen was adjusted to oral pyrotinib (400mg, once daily) plus capecitabine (1000mg/m^2^, twice daily on days 1–14 of each 21-day cycle).

Beginning in May 2022, chest computed tomography (CT) showed a significant increase in left-sided pleural effusion. Thoracentesis and cytological examination confirmed pleural metastasis of breast cancer, indicating disease progression. From August 2017 to May 2022, the patient continuously received anti-HER2 treatment, and the PFS reached 57 months. IHC analysis of pleural fluid showed CK (+), GATA-3 (+), ER (80%, varying intensity), PR (10% weak +), HER2 (2+), CK5/6 (−), CR (−), TTF-1 (−), and Ki-67 proliferation index of 80%, no amplification by FISH. Based on this profile suggesting a highly proliferative, HR+ phenotype, the treatment regimen was switched to fulvestrant (500 mg, once every 28 days) plus abemaciclib (150 mg, twice daily). The disease is under stable control.

In January 2024, chest CT again indicated an increase in pleural effusion compared to before. Thoracentesis and drainage were performed, and pathological analysis of the pleural fluid again supported metastatic breast cancer. IHC demonstrated CK (+), GATA-3 (+), TRPS1 (partially +), ER (40% weak-moderate +), PR (−), HER2 (2+), TTF-1 (−), Napsin A (−), CR (−), Pax-8 (−), and Ki-67 proliferation index of 40%, with HER2 amplification detected by FISH. At the same time, a dose of 40 mg of cisplatin was administered via intrathoracic injection for therapeutic purposes. Simultaneously, treatment with DS-8201 (5.4 mg/kg, once every 21 days) was started. A total of eight cycles were completed, during which efficacy evaluations after the second and fourth cycles showed stable disease.

**Figure 1:**
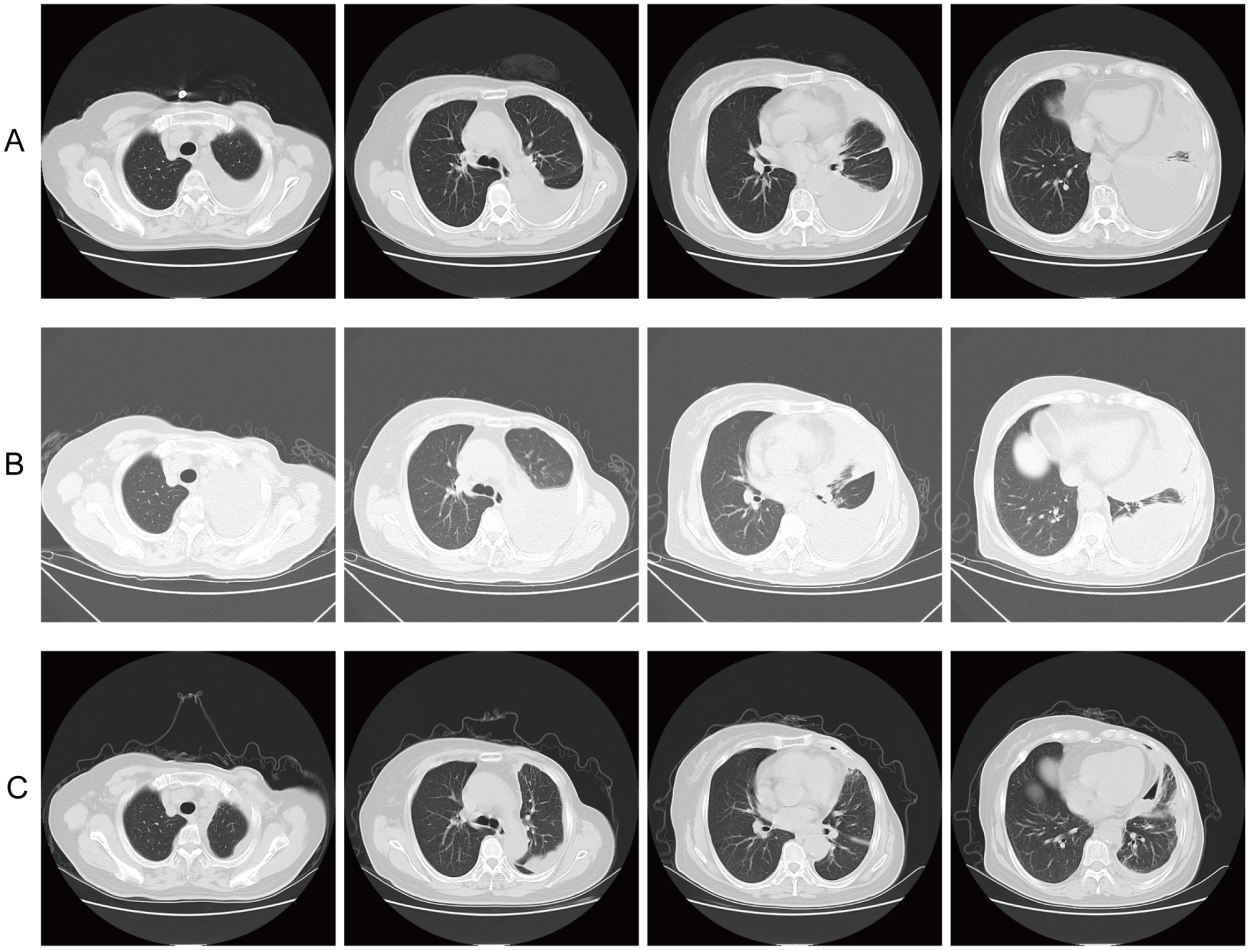
Serial CT imaging of left pleural effusion across three treatment phases at four anatomical levels. **(A)** Baseline (August 2024): Extensive pleural effusion before treatment with DS-8201 plus intrapleural cisplatin. **(B)** Post-cisplatin (October 2024): Increased pleural effusion after 3 cycles of DS-8201 plus intrapleural cisplatin. **(C)** Post-bevacizumab (November 2024 onward): Marked reduction of pleural effusion after switching to DS-8201 plus intrapleural bevacizumab.

In August 2024, IHC analysis of the pleural fluid showed ER (40% weak-moderate +), PR (−), and HER2 (1+). Subsequently, from August to October, on the basis of continuing the DS-8201 treatment, combined with intrapleural cisplatin (40mg, once every 21 days) treatment for three cycles. However, the pleural effusion did not significantly decrease. By October 2024, chest CT demonstrated further progression of pleural effusion. Thoracentesis and cytopathology reaffirmed metastatic breast cancer, with IHC showing CK7 (+), TRPS1 (−), GATA-3 (+), ER (40% moderate-strong +), PR (−), HER2 (1+), CR (−), CK5/6 (−), TTF-1 (−), and Ki-67 proliferation index of approximately 40%. Considering the poor response to cisplatin-based combination therapy and the theoretical rationale for anti-angiogenic therapy in controlling vascular permeability, the therapeutic strategy was adjusted in November 2024 to DS-8201(5.4 mg/kg, once every 21 days) plus intrapleural bevacizumab (200 mg, once every 21 days). After three cycles, chest CT showed a significant decrease in pleural effusion (**Figure 1**).

**Figure 2:**
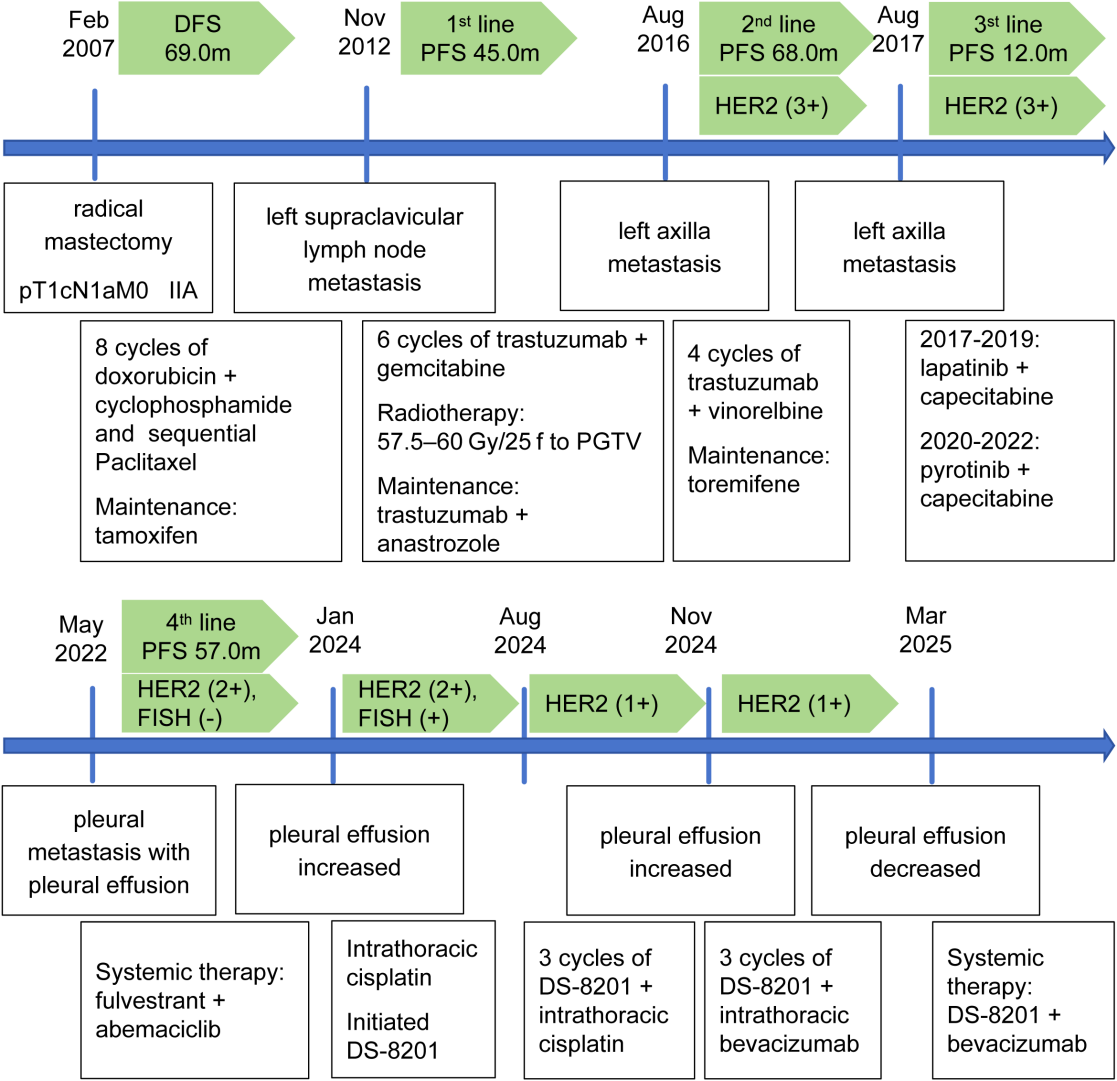
Timeline of disease progression and treatment in the patient.

Based on the marked reduction of pleural effusion achieved with the previous combination of DS-8201 and intrapleural bevacizumab, the therapeutic strategy was escalated in March 2025 to systemic treatment with DS-8201 (5.4 mg/kg, once every 21 days) and bevacizumab (10 mg/kg, once every 21 days) to consolidate and extend the treatment effect. During follow-up, the pleural effusion remained well-controlled and the disease remained stable. Throughout the course of treatment, no adverse effects related to DS-8201 or bevacizumab were observed. During the entire course of treatment with DS-8201 and bevacizumab, the patient demonstrated good tolerance, with no significant drug-related adverse events observed. Common adverse effects associated with DS-8201, such as gastrointestinal symptoms (e.g., nausea, vomiting), hematologic toxicities (e.g., neutropenia, anemia), alopecia, or interstitial lung disease, were not reported. Likewise, no bevacizumab-related adverse events, including hypertension, proteinuria, bleeding tendency, or venous thromboembolism, were observed. There was no need for treatment interruption or dose modification due to toxicity, and the patient’s quality of life was notably improved (**Figure 2**).

## Discussion

**Figure 3:**
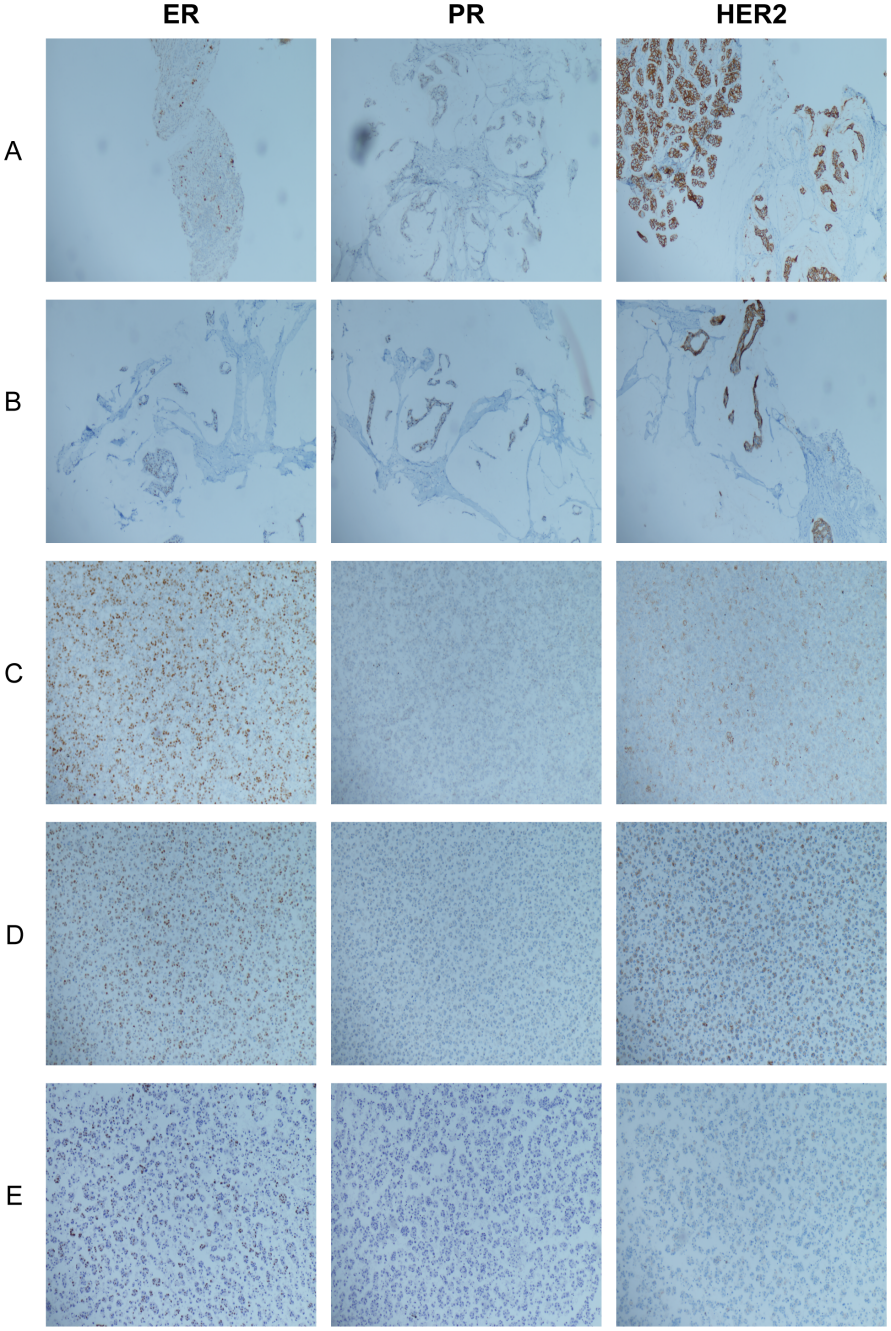
Dynamic changes in Estrogen Receptor (ER), Progesterone Receptor (PR), and Human Epidermal Growth Factor Receptor 2 (HER2) expression during disease progression. **(A)** In August 2016, left axillary lymph node tissue: ER (10%+), PR (approximately 2% weak+), HER2 (3+); **(B)** In August 2017, left axillary lymph node tissue: ER (approximately 20% moderate-strong+), PR (approximately 30% weak-moderate+), HER2 (3+); **(C)** In May 2022, pleural effusion cytological specimen: ER (80%, varying intensity), PR (10% weak+), HER2 (2+); **(D)** In January 2024, pleural effusion cytological specimen: ER (40% weak-moderate+), PR (−), HER2 (2+); **(E)** In August 2024, pleural effusion cytological specimen: ER (40% weak-moderate+), PR (−), and HER2 (1+).

This patient is a female with a 17-year history of breast cancer. No distant metastasis was observed at initial diagnosis. The patient subsequently received a series of standardized comprehensive therapies, including surgery, chemotherapy, radiotherapy, endocrine therapy, and targeted therapy, which achieved long-term disease control and maintained a good standard of life. In the latter stages of the disease, patients mainly present with persistent recurrent malignant pleural effusion. After the occurrence of pleural effusion, the patient briefly received DS-8201 combined with pleural perfusion of cisplatin treatment, but the clinical benefit was limited, with poor control of the pleural effusion and minimal symptom relief. Considering the key role of tumor-driven vascular leakage in effusion formation, the treatment plan was further adjusted to DS-8201 plus bevacizumab therapy. This combined plan rapidly improved pleural effusion, significantly alleviated discomfort and enhanced the standard of life. During the treatment process, monitoring revealed that the expression level of HER2 showed a gradually decreasing trend, successively decreasing from the initial IHC 3+ to 2+ and 1+, and was eventually confirmed as HER2-low breast cancer (**Figure 3**). The variation in HER2 expression may be influenced by differences in sampling sites, particularly as tumor cell subpopulations identified in pleural effusion cell block specimens may differ from those in the primary tumor, potentially leading to discrepancies in test results. Furthermore, the dynamic changes in HER2 expression may also be closely related to the enhanced tumor heterogeneity such as tumor clonal evolution and molecular phenotypic drift after multi-line systematic treatment. Increased tumor heterogeneity not only alters therapeutic targets but also promotes the coexistence and evolution of various subclones, some of which may exhibit higher invasive and metastatic potential, thereby facilitating pleural dissemination and the development of malignant pleural effusion. These dynamic changes suggest that tumor cells may progressively lose HER2 dependence during long-term treatment, shifting reliance to alternative signaling pathways to sustain survival and progression, which in turn increases the complexity and challenges of disease management. Collectively, these factors underscore the importance of re-biopsy at progression to assess the current tumor biology and guide precise therapy.

HER2-low breast cancer has been increasingly identified as a distinct therapeutic subtype in recent years. Its treatment model mainly benefits from the rapid development of novel ADCs, such as DS-8201 and sacituzumab govitecan, providing new treatment hope for this type of patients. DS-8201 is an HER2-targeted ADC that carries a potent topoisomerase I inhibitor DXd (12). It features a high drug-antibody ratio (DAR) and a “bystander effect” (13). Thanks to its unique mechanism of action, it has demonstrated outstanding anti-tumor efficacy and controllable safety in multiple clinical studies and has been successively approved for the management of HER2-positive and HER2-low expression breast cancer (3, 14–16). In HER2-positive breast cancer, the latest DESTINY-Breast03 trial (17) demonstrated that DS-8201 significantly outperformed T-DM1, with a median PFS of 29.0 months over 7.2 months for the T-DM1 group. Median OS was also extended by nearly one year (52.6 months vs. 42.7 months, HR = 0.73;95%CI 0.56-0.94). This study redefined the treatment criteria for HER2-positive breast cancer and established the core position of DS-8201 in this population. The DESTINY-Breast04 trial was the first study to confirm the efficacy of DS-8201 in patients with HER2-low breast cancer. Among all patients with HER2-low, the median PFS of DS-8201 was 9.9 months, which was significantly better than 5.1 months in the chemotherapy cohort (HR = 0.50; 95% CI 0.40–0.63),and the median OS was also significantly extended (23.4 months vs. 16.8 months; HR = 0.64; 95% CI 0.49–0.84). In subgroup of HR-positive and HER2-low breast cancer, the median PFS with DS-8201 was 10.1 months, significantly longer than with chemotherapy (5.4 months; HR = 0.51, 95%CI: 0.40-0.64). Median OS was also prolonged (23.9 months vs. 17.5 months; HR = 0.64, 95% CI: 0.48-0.86). The subsequent DESTINY-Breast06 trial further reinforced the therapeutic position of DS-8201 in patients with HR+, HER2-low or HER2-ultralow breast cancer. This study shows that the median PFS in DS-8201 cohort reached 13.2 months, which was 5.1 months longer than 8.1 months in the control cohort (HR = 0.63;95% CI 0.53-0.75). This result supports the extension of DS-8201 from the previous late-line treatment to the more advanced treatment stage, and it has become an important therapeutic option for advanced breast cancer characterized by HR+, HER2-low or HER2-ultralow status subsequent to endocrine therapy failure. Based on the above research results, DS-8201 has shown consistent and significant efficacy in patients with HER2-low breast cancer, especially among those who have underwent at least one line of chemotherapy in the past, and has become the standard treatment option for this group of people. However, for HER2-low breast cancer patients whose primary presentation is malignant pleural effusion, there is still a lack of targeted treatment strategies at present. While DS-8201 has reshaped the treatment landscape for advanced HER2-low breast cancer, this case compels us to reevaluate early-stage management strategies. The absence of trastuzumab therapy in the early stages may fundamentally alter long-term prognosis by facilitating early metastatic spread. Therefore, ensuring equitable access to guideline-directed curative treatments remains crucial for preventing complex advanced disease.

The rapid development of ADCs has broadened their role in breast cancer, showing particular promise in HER2-low disease. Yet, tumor microenvironment complexity and resistance evolution limit monotherapy, prompting exploration of combination strategies. In HER2-positive disease, trials such as DESTINY-Breast07/09 and HER2CLIMB-02/04 have tested DS-8201 or T-DM1 with agents like pertuzumab or tucatinib, aiming to boost ADC internalization and antitumor activity. In HER2-low, studies like TALENT and DESTINY-Breast08 combined DS-8201 with endocrine therapy, while other trials (e.g., KATE2, BEGONIA, DS8201-A-U105) investigated combinations with immunotherapy, PI3K, or CDK4/6 inhibitors. Increasing attention is now on pairing ADCs with anti-angiogenic therapy. Supported by mechanistic rationale and early evidence, we applied DS-8201 plus bevacizumab in this case to better control malignant pleural effusion and enhance the antitumor response (**Table 1**).

**Table 1:** Ongoing and completed clinical trials of ADC combination therapies in breast cancer.

Category	Trial name	Target population	Combination regimen	Mechanism/Purpose	Phase/Status
HER2-Positive Breast Cancer	DESTINY-Breast07	HER2+	DS-8201 + Pertuzumab	Enhanced ADC internalization and antitumor activity	Phase III (Ongoing)
	DESTINY-Breast09	HER2+	DS-8201 + Pertuzumab + Docetaxel	First-line regimen combining chemotherapy with dual HER2-targeted therapy	Phase III (Ongoing)
	HER2CLIMB-02	HER2+ (incl. brain mets)	Tucatinib + T-DM1	HER2 pathway inhibition combined with cytotoxic drug delivery via ADC	Phase III (Positive)
	HER2CLIMB-04	HER2+	Tucatinib + DS-8201	Dual HER2 blockade with bystander effect from topoisomerase I inhibitor payload	Phase II (Recruiting)
HER2-Low Breast Cancer	TALENT	HER2-low/HR+	DS-8201 + Fulvestrant	ADC therapy combined with endocrine therapy to overcome hormone receptor resistance	Phase II (Preliminary)
	DESTINY-Breast08	HER2-low/HR+	DS-8201 + Anastrozole/Letrozole	ADC therapy combined with estrogen suppression to enhance efficacy in HR+/HER2-low breast cancer	Phase Ib/II (Ongoing)
Combination with Immunotherapy	KATE2	HER2+ (PD-L1+)	T-DM1 + Atezolizumab	Immunogenic cell death induced by ADC combined with PD-L1 blockade	Phase II (Negative)
	BEGONIA(DS-8201 cohort)	TNBC/HER2-low	DS-8201 + Durvalumab	Topoisomerase I inhibitor payload combined with PD-L1 blockade to enhance antitumor immune response	Phase II (Ongoing)
Combination with Targeted Therapy	DS8201-A-U105	HER2+/HER2-low	DS-8201 + Olaparib (PARP inhibitor)	Synergistic DNA damage through PARP inhibition and topoisomerase I inhibitor payload	Phase I/II (Ongoing)

ADC, antibody–drug conjugate; HER2, human epidermal growth factor receptor 2; HR, hormone receptor; PARP, poly(ADP-ribose) polymerase; PD-L1, programmed cell death ligand 1; TNBC, triple-negative breast cancer.

Bevacizumab, as a representative drug for anti-angiogenic therapy, targets the VEGF signaling pathway to reduce tumor-mediated vascular leakage, thereby effectively controlling vascular leakage caused by tumors and achieving the goal of controlling pleural effusion. In addition, its mechanism of “vascular normalization” may improve the local hypoxic microenvironment within tumors, enhance the penetration and delivery of anti-tumor drugs, and play a pivotal role in mitigating increased vascular permeability and the formation of malignant effusions (18). Moreover, bevacizumab contributes to the remodeling of the tumor microenvironment by promoting immune cell infiltration and alleviating immunosuppression, further enhancing anti-tumor efficacy. In this case, the patient had previously received a regimen of DS-8201 combined with intrapleural cisplatin infusion, which failed to achieve adequate control of pleural effusion. Following a regimen switch to DS-8201 combined with bevacizumab, the pleural effusion was rapidly relieved, symptoms were significantly improved, and quality of life was markedly enhanced. It is speculated that its therapeutic effect may stem from the multiple synergistic mechanisms of “drug delivery optimization, microenvironment remodeling and immune activation”, showing a better comprehensive therapeutic effect than the traditional chemotherapy combination regimen.

In terms of safety, the patient demonstrated good overall tolerability throughout the entire course of DS-8201 combined with bevacizumab therapy, with no significant drug-related serious adverse events observed. Common side effects associated with DS-8201, such as myelosuppression and gastrointestinal reactions, as well as bevacizumab-related hypertension and proteinuria, were not prominently manifested in this case, allowing the patient’s quality of life to be maintained. Prior to initiating DS-8201 plus bevacizumab, all treatment regimens were formulated in accordance with prevailing clinical guidelines and evidence-based practice, ensuring adherence to standardized therapeutic principles. Given the suboptimal control of recurrent pleural effusion, DS-8201 was combined with bevacizumab based on the complementarity of their mechanisms, which led to improved effusion control and symptom relief, illustrating a potential treatment approach for complex clinical scenarios.

## Conclusion

In summary, DS-8201 plus bevacizumab may be a promising option for HER2-low breast cancer with malignant pleural effusion. However, as a single-case observation without a control group, these findings are preliminary. Therefore, prospective clinical trials are warranted to rigorously evaluate the efficacy and safety of this combination therapy.

## Data Availability

The original contributions presented in the study are included in the article/**Supplementary Material**. Further inquiries can be directed to the corresponding authors.
