# Supplementary material for: Trastuzumab deruxtecan (DS-8201) plus bevacizumab for HER2-low metastatic breast cancer with malignant pleural effusion: a case report and literature review
Source: Front Oncol. 2026 Jan 12;15:1694553. doi: 10.3389/fonc.2025.1694553 (PMC12832279; doi:10.3389/fonc.2025.1694553)
Supplement: Supplementary file 1 [file DataSheet1.pdf]

CARE Checklist of information to include when writing a case report

CARE case report guidelines

(cc) BY-NC-ND

Topic	Item	Checklist item description	Reported on Line
Title	1	The diagnosis or intervention of primary focus followed by the words "case report"	1-3
Key Words	2	2 to 5 key words that identify diagnoses or interventions in this case report, including "case report"	14
Abstract (no references)	3a	Introduction: What is unique about this case and what does it add to the scientific literature?	16-19
	3b	Main symptoms and/or important clinical findings	20-25
	3c	The main diagnoses, therapeutic interventions, and outcomes	26-32
	3d	Conclusion—What is the main "take-away" lesson(s) from this case?	33-36
Introduction	4	One or two paragraphs summarizing why this case is unique (**may include references**)	37-78
Patient Information	5a	De-identified patient specific information.	80-81
	5b	Primary concerns and symptoms of the patient.	80-81
	5c	Medical, family, and psycho-social history including relevant genetic information	81-82
	5d	Relevant past interventions with outcomes	Figure2
Clinical Findings	6	Describe significant physical examination (PE) and important clinical findings.	125-126
Timeline	7	Historical and current information from this episode of care organized as a timeline	Figure2
Diagnostic Assessment	8a	Diagnostic testing (such as PE, laboratory testing, imaging, surveys).	85/125/130/143
	8b	Diagnostic challenges (such as access to testing, financial, or cultural)	-
	8c	Diagnosis (including other diagnoses considered)	176-179
	8d	Prognosis (such as staging in oncology) where applicable	155-157
Therapeutic Intervention	9a	Types of therapeutic intervention (such as pharmacologic, surgical, preventive, self-care)	149-154
	9b	Administration of therapeutic intervention (such as dosage, strength, duration)	149-154
	9c	Changes in therapeutic intervention (with rationale)	149-154
Follow-up and Outcomes	10a	Clinician and patient-assessed outcomes (if available)	157-164
	10b	Important follow-up diagnostic and other test results	152/157-164
	10c	Intervention adherence and tolerability (How was this assessed?)	157-164
	10d	Adverse and unanticipated events	157-164
Discussion	11a	A scientific discussion of the strengths AND limitations associated with this case report	166-260
	11b	Discussion of the relevant medical literature **with references**.	191-249
	11c	The scientific rationale for any conclusions (including assessment of possible causes)	191-198/234-249
	11d	The primary "take-away" lessons of this case report (without references) in a one paragraph conclusion	262-263
Patient Perspective	12	The patient should share their perspective in one to two paragraphs on the treatment(s) they received	250-252
Informed Consent	13	Did the patient give informed consent? Please provide if requested	**Yes**
